# Hot-Cracking Mechanism of Laser Welding of Aluminum Alloy 6061 in Lap Joint Configuration

**DOI:** 10.3390/ma16196426

**Published:** 2023-09-27

**Authors:** Km Rakhi, Seunggu Kang, Joonghan Shin

**Affiliations:** 1 Department of Future Convergence Engineering, Kongju National University, 1223-24 Cheonandaero, Seobuk-gu, Cheonan 31080, Republic of Korea; rakhi@smail.kongju.ac.kr (K.R.); topkun97@gmail.com (S.K.); 2 Department of Mechanical and Automotive Engineering, Kongju National University, 1223-24 Cheonandaero, Seobuk-gu, Cheonan 31080, Republic of Korea; 3 Global Institute of Manufacturing Technology (GITECH), Kongju National University, 1223-24 Cheonandaero, Seobuk-gu, Cheonan 31080, Republic of Korea

**Keywords:** laser welding, aluminum alloy 6061 (AA6061), lap joint, cracking susceptibility, hot-cracking mechanism

## Abstract

Laser welding, known for its distinctive advantages, has become significantly valuable in the automotive industry. However, in this context, the frequent occurrence of hot cracking necessitates further investigation into this phenomenon. This research aims to understand the hot-cracking mechanism in aluminum alloy (AA) 6061, welded using a laser beam in a lap joint setup. We used an array of material characterization methods to study the effects of processing parameters on the cracking susceptibility and to elucidate the hot-cracking mechanism. A laser power of 2000 W generated large hot cracks crossing the whole weld zone for all welding speed conditions. Our findings suggest that using a heat input of 30 J/mm significantly mitigates the likelihood of hot cracking. Furthermore, we observed that the concentrations of the alloying elements in the cracked region markedly surpassed the tolerable limits of some elements (silicon: 2.3 times, chromium: 8.1 times, and iron: 2.7 times, on average) in AA6061. The hot-cracking mechanism shows that the crack initiates from the weld root at the interface between the two welded plates and then extends along the columnar dendrite growth direction. Once the crack reaches the central region of the fusion zone, it veers upward, following the cooling direction in this area. Our comprehensive investigation indicates that the onset and propagation of hot cracks are influenced by a combination of factors, such as stress, strain, and the concentration of alloying elements within the intergranular region.

## 1. Introduction

The aluminum 6061 alloy is favored in a variety of industries, including the automotive, marine, and aerospace industries, owing to its exceptional mechanical properties and superior corrosion resistance compared to other aluminum alloy series [1,2,3]. Numerous fusion-based welding techniques are employed to join aluminum alloys, among which laser welding is a promising method [4,5,6]. However, laser welding of these alloys often results in defects such as voids and hot cracks [7,8,9]. Hot cracking, in particular, is a critical defect likely to occur during the laser welding of aluminum alloys [10,11]. The chemical composition of these alloys increases their vulnerability to hot cracking [12]. Specifically, the morphology of the weld zone considerably influences the formation of hot cracks.

The susceptibility to hot cracking and its underlying mechanisms have been thoroughly studied by many researchers. Rappaz et al. [13] created a numerical model by considering various microstructural and thermal parameters such as the grain morphology, size, and orientation as well as the temperature field and the macroscopic strain rate. This model offers insights into the genesis of hot cracks. Kou et al. [14] demonstrated that hot cracking is more likely to occur within a partially melted zone owing to an increased vulnerability to the tensile stresses and strains generated during solidification. This is attributed to the formation of residual stresses during the phase transition from liquid to solid, which can lead to cracking if the yield strength of the material is surpassed. Liu et al. [15] introduced a thermomechanical model by considering the movement of impurities within a material, influencing its mechanical and thermal properties, and employed strain theory to assess the cracking susceptibility of a material. Wang et al. [16] formulated a finite element model that simulated aluminum alloy behavior during laser welding, emphasizing strain localization in the mushy transitional zone. This model predicts the likelihood of cracking during welding by evaluating the local deformation of the material. Various other hot-cracking models and criteria have been explored and analyzed in the referenced studies [17,18,19,20,21].

Despite numerous studies on hot cracking during the laser welding of aluminum alloys, there is a need for additional research. Most of these studies have concentrated on the butt joint configuration. In contrast, lap joint laser welding—common when joining structural components such as automotive chassis or body panels—remains less explored. This technique, employed with alloys such as AA6061, facilitates lightweight construction and a high strength, stiffness, and corrosion resistance. Thus, it is critical to comprehend the mechanisms of hot cracking within this context, and further research is needed to devise effective prevention strategies.

In this work, we aimed to study the hot-cracking mechanism for the laser lap joint welding of AA6061. A series of experiments were designed and conducted to generate both sound (without hot cracking) and defective (with hot cracking) welds. The effects of the processing conditions on the cracking susceptibility were investigated, and various material characterization methods were used to elucidate the hot-cracking mechanism. It is expected that the proposed hot-cracking mechanism in this study contributes to enhancing the understanding of the hot-cracking phenomenon in the laser welding of AA6061 under a lap joint configuration.

## 2. Material and Methods

### 2.1. Material

The experimental samples were rectangular plates of AA6061, each 2 mm thick and artificially aged to the T6 condition (AA6061-T6). The dimensions of each plate were 70 mm in length and 40 mm in width. Prior to welding, the specimen surfaces were meticulously cleaned with acetone to prevent any surface contamination issues. Table 1 provides the elemental composition of AA6061.

### 2.2. Experimental Procedure

**Figure 1:**
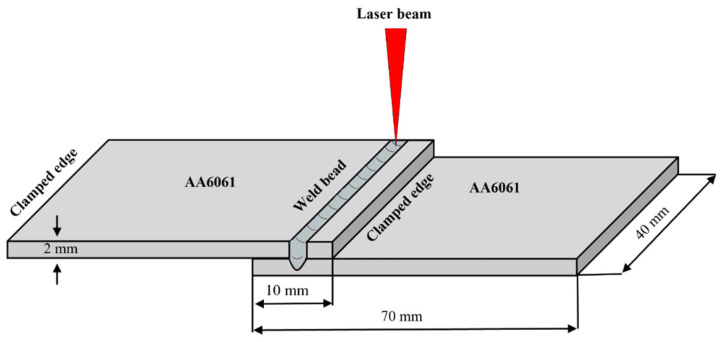
Schematic of lap joint weld configuration.

This study focuses on the laser lap welding of an aluminum alloy (AA6061) using a single-mode continuous-wave ytterbium fiber laser system (Raycus, RFL-C2000). This system delivers a maximum power output of 2000 W at a wavelength of 1080 nm. The laser beam, transmitted through an optical fiber, was focused onto the surface of the specimen using a focusing lens with a focal length of 200 mm. The diameter of the beam spot on the workpiece surface was 100 μm. Full penetration of the laser beam into the top plate is required during the laser lap welding process to ensure the complete fusion of the base metal. To achieve this, we identified suitable process conditions via bead-on-plate experiments to guarantee the complete penetration of the laser beam into the AA6061 plate. Table 2 presents the optimal process conditions. To maintain uniformity and limit distortion and residual stress variations, the plates were secured during the welding experiment. Figure 1 depicts a schematic of the lap joint setup and the clamping conditions.

### 2.3. Sample Preparation for Weld Morphology and Microstructure Analysis

Following the welding experiment, the welded samples were sectioned perpendicular to the welding direction and secured using a fixation clip, in conjunction with a mixture of epoxy resin and a hardener, a method known as cold mounting. The secured samples were then subjected to grinding with sandpaper and polishing with a diamond suspension up to 1 μm utilizing a polishing cloth. To facilitate the observation of the weld morphology and conduct a microstructural analysis, the polished surfaces of the samples were chemically etched using Keller’s reagent, composed of 5 mL of NO_3_, 3 mL of HCL, 2 mL of HF, and 20 mL of H_2_O. The surface morphologies of the weld bead and weld cross-section were scrutinized using optical microscopy (OM; Dino Lite, Torrance, CA, USA, AM4013MZT) and scanning electron microscopy (SEM; TESCAN, Warrendale, PA, USA, VEGA3). To measure the top weld bead width, five points along the welding direction were selected (each 2.5 mm apart on the image), and the average value from these measurements determined the bead width. An elemental composition analysis of the weld fusion zone (FZ) was performed using energy-dispersive X-ray spectroscopy (EDS; BRUKER, Billerica, MA, USA, flash6130). The microstructure, grain morphology, and grain size were examined using electron backscatter diffraction (EBSD; HIKARI SUPER, Nagoya, Japan, SU5000), with measurements acquired under a 20 kV electron and a step size of 2 μm.

## 3. Results and Discussion

### 3.1. Analysis of Weld Morphology and Hot Cracking

**Figure 2:**
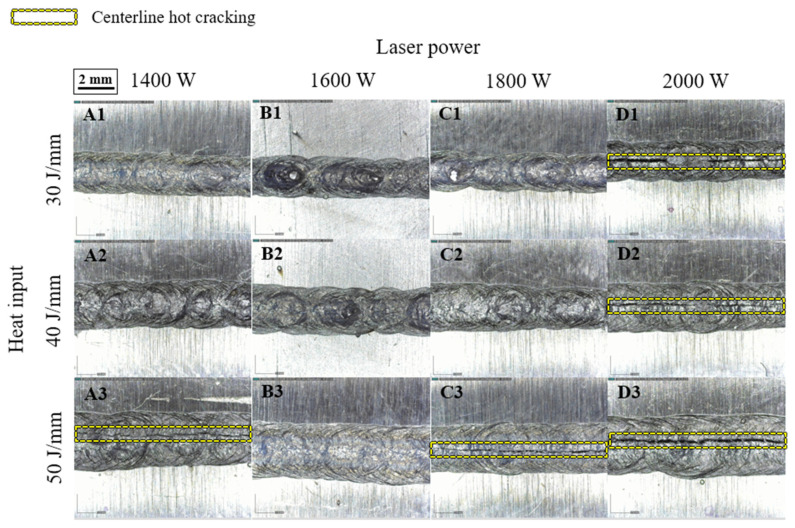
Top surface images of the weld zone.

Figure 2 presents optical micrographs of the top weld beads from various weld joints, exhibiting varying widths under distinct heat inputs. Specifically, the width tended to expand with increasing laser power, but it contracted with increasing welding speed. This relationship among weld bead width, laser power, and welding speed is likely due to heat input variations influenced by parameter adjustments. Upon the OM examination of the weld joint, centerline hot cracking was found to occur at a relatively high heat input (50 J/mm). Centerline hot cracking was the most pronounced in samples A3, C3, D1, D2, and D3, while no visible cracks were identified on the weld beads of the remaining samples. For samples D1 and D2, hot cracking primarily stemmed from the relatively high power density (2.54 × 10^5^ W/mm^2^), given that the heat input was comparatively low. Conversely, for samples A3, C3, and D3, hot cracking was primarily due to a high heat input (50 J/mm), which created a higher thermal gradient in the material, leading to increased residual stresses and strains and resulting in hot cracking. The sample D3, welded with the highest power density (2.54 × 10^5^ W/mm^2^), showed the largest crack width (~160 μm); however, the crack width decreased to ~60 μm when the lowest power density (A3: 1.78 × 10^5^ W/mm^2^) was applied. Overall, the susceptibility to hot cracking was found to decrease as the heat input was reduced. Apart from the heat input, the interaction time (the duration of laser beam exposure to the material) can also impact hot cracking; a longer interaction time implies more energy absorption by the material, which escalates the risk of hot cracking. Based on the above analysis and the data presented in Figure 2, we concluded that the welding process parameters for the samples with a laser power of 1600 W were not prone to centerline hot cracking, as no such cracking was observed in samples B1, B2, or B3.

**Figure 3:**
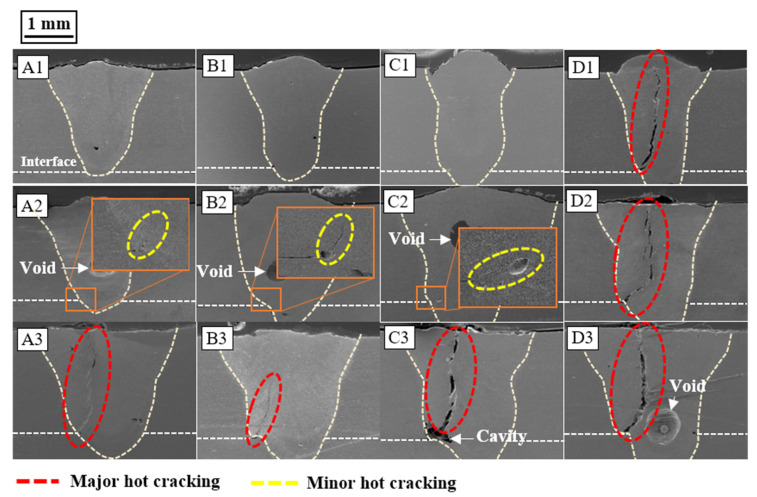
Scanning electron microscopy (SEM) cross-section images of the weld zone.

**Figure 4:**
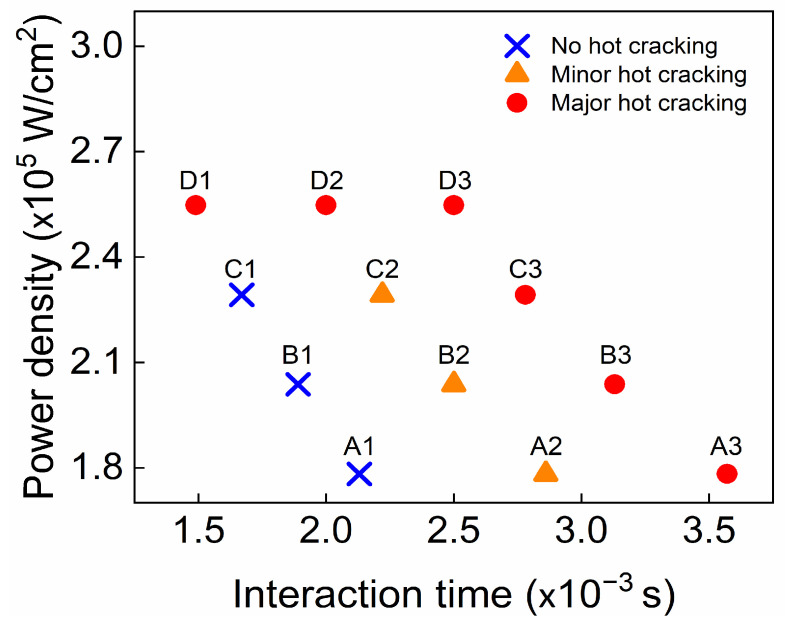
Effect of power density and interaction time on hot cracking.

Figure 3 presents SEM-generated cross-sectional images of the welds, showing fully formed welds in AA6061. An examination of these cross-sections revealed the formation of cracks and voids within the welds. These findings suggest that the penetration depth escalates with increased heat input and laser power, while it diminishes with increasing welding speed. Notably, the cracks were found to originate from the fusion boundary at the interface between the upper and lower plates, propagating towards the FZ center. This occurrence is likely due to the highest thermal stresses being associated with solidification shrinkage near the fusion line, and these stresses can be relayed towards the weld pool center. Upon reaching the center of the FZ, the cracks proceeded to propagate upward along the centerline of the weld. Hot cracking was most conspicuous in the samples with a high heat input (50 J/mm: A3, B3, C3, and D3) and a high power density (2.54 × 10^5^ W/mm^2^: D1 and D2). In these cases, the crack length was in the range of ~1.1 to ~2.3 mm. Mild cracking was observed in the samples with a moderate heat input (40 J/mm: A2, B2, and C2), whereas no visible cracks were detected at a low heat input (30 J/mm: A1, B1, and C1). In the case of mild cracking, the measured crack length was in the range of ~130 to ~270 μm. Overall, the susceptibility to hot cracking declined as the heat input decreased. Figure 4 additionally illustrates the impact of the power density and interaction time on hot cracking, demonstrating the relationship between hot cracking and the process parameters. As Figure 4 depicts, the combination of a high power density and an extended interaction time was more susceptible to hot cracking. As this combined value diminished, the susceptibility to hot cracking reduced correspondingly. A high power density implies that a high energy is concentrated on a smaller area for a shorter duration, leading to higher temperatures and faster heating and solidification rates. The rapid heating and cooling rates linked with a high power density generate significant thermal gradients within the metal, resulting in high residual stresses in the solidifying metal that foster hot cracking. An extended interaction time also leads to a high temperature and thermal stress concentration, which in turn promotes hot cracking.

### 3.2. Analysis of Microstructure by EBSD

**Figure 5:**
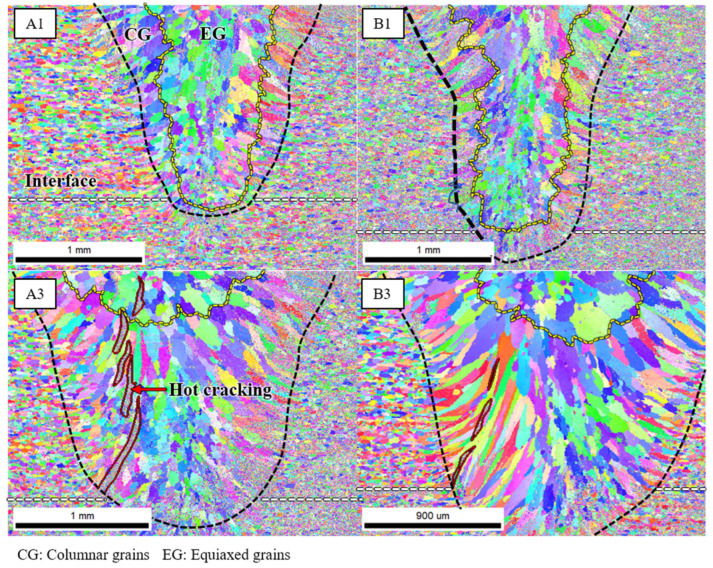
Electron backscatter diffraction (EBSD) inverse pole figure (IPF) maps for transversal cross-section of the weld.

An EBSD analysis was performed to examine the microstructural features of the welds. Figure 5 displays the inverse pole figure (IPF) maps for the transverse cross-section of the weld zone for samples A1, A3, B1, and B3. As presented in Figure 5, the microstructure of the weld zone varied significantly from that of the base metal. Specifically, the grains were finer for a heat input of 30 J/mm compared to 50 J/mm. This observation was ascribed to the higher welding speed associated with a lower heat input and a slower welding speed for a higher heat input. When the welding speed was high, it resulted in an accelerated cooling rate, supporting faster cooling and the formation of smaller grains. Furthermore, a lower heat input implied less heat being applied to the base metal, preventing it from reaching elevated temperatures and further facilitating the formation of finer grains. In the weld zone, columnar grains were predominantly situated at the boundary of the weld, whereas equiaxed grains were largely distributed at the center of the weld. This arrangement resulted from the solidification process of the weld, with columnar grains initially growing from the weld boundary when the weld boundary temperature was at its lowest. These grains persistently grew towards the weld center until the new nucleation of equiaxed grains occurred in the remaining central melt pool. Hence, the solidification process determined the grain distribution. Moreover, Figure 5 indicates that samples A1 and B1 (with a heat input of 30 J/mm) did not present hot cracking, while samples A3 and B3 (with a heat input of 50 J/mm) displayed hot cracking. This phenomenon can be elucidated by the differences in the grain growth mechanisms triggered by varying welding conditions. Solidification, contributing to the formation of larger grains, was implicated in the growth of hot cracks. During solidification, large grains possessed larger intergranular regions. Consequently, they harbored fewer grain boundaries, which created structurally weak areas. The stress concentration was elevated in these intergranular regions, making them act as a path for crack growth. As depicted in Figure 5, hot cracks were apparent in the intergranular regions. After hot cracking, the cracks tracked the columnar grain boundaries towards the center, ultimately propagating in an upward direction.

**Figure 6:**
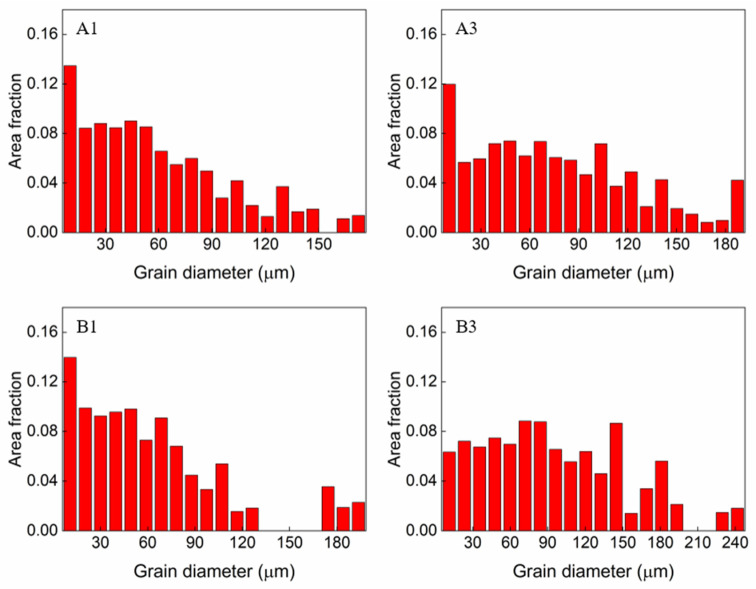
Area fraction of grain size as a function of grain diameter.

Figure 6 depicts the fraction of the grain area as a function of the grain diameter. The average grain size for the samples under a low heat input condition (30 J/mm) was 91 µm for A1 and 96 µm for B1, whereas for the samples under a high heat input condition (50 J/mm), it was 93 µm for A3 and 126 µm for B3. Based on the aforementioned analysis, it can be deduced that the grain size expands with increasing heat input. Consequently, the samples subjected to a high heat input and exhibiting large grain sizes experienced significant hot cracking.

### 3.3. EDS Analysis of Chemical Composition

**Figure 7:**
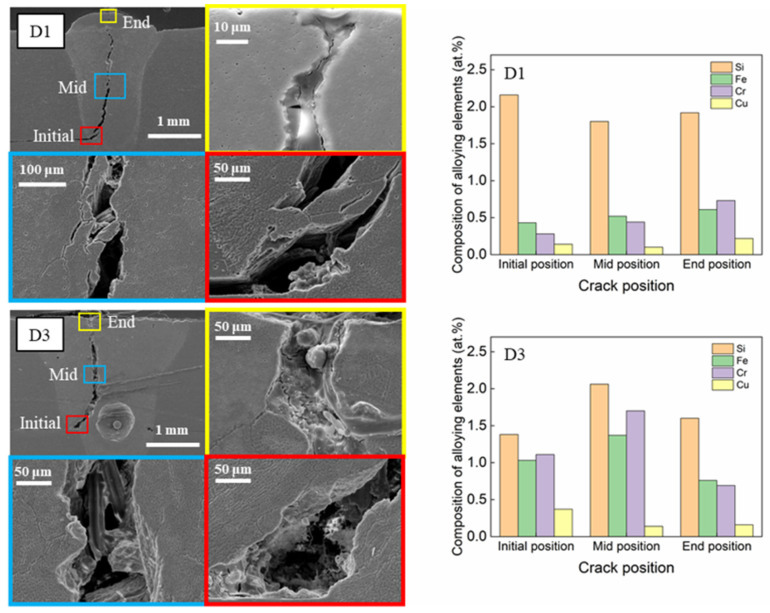
Results of energy-dispersive X-ray spectroscopy (EDS) area mapping at different crack positions for samples D1 and D3.

Using the EDS area and point mapping, the concentrations of the alloying elements in the selected samples were measured. According to the EDS analysis, an instrumental error for the EDS analysis was usually less than 0.1%. Therefore, in this study, we neglected the instrumental error in displaying the results of the EDS analysis. Figure 7 displays the results of the EDS area mapping for samples D1 and D3, both of which exhibited significant hot cracking due to the use of the highest power density (2.54 × 10^5^ W/mm^2^). The data show a notably high concentration of silicon (Si) in both samples. For sample D1, the atomic percentages (at. %) of Si were 2.16%, 1.80%, and 1.92% at the initial, middle, and end stages of hot-crack formation, respectively. Similarly, for D3, the Si concentrations were measured as 1.38%, 2.06%, and 1.60% at the same stages. Additionally, the quantities of chromium (Cr) and iron (Fe) were markedly higher in sample D3 than in D1, likely due to a higher heat input during welding. A high heat input implies a slow welding speed (i.e., low solidification rate), allowing a longer period for the alloying elements to segregate in the weld pool. Due to their lower diffusivity in Al, these elements move more slowly with the solidification front compared to Al and become trapped in the interdendritic region. As the weld solidifies, these alloying elements are gradually forced toward the center of the pool as the solidification front moves from the fusion boundary to the center. Conversely, under conditions of a low heat input (i.e., high welding speed), the alloying elements have a comparatively shorter time to segregate throughout the weld, leading to lower concentrations in the samples subjected to a lower heat input compared to those with a higher heat input. However, the copper (Cu) concentration did not show a significant variation. Interestingly, Figure 7 indicates no detectable levels of magnesium (Mg) in samples D1 and D3, likely due to the high vaporization of Mg, which has a relatively low boiling point (1091 °C). This is especially pronounced near the cracks, as they typically form along the centerline, the region with the highest temperature in the weld zone.

**Figure 8:**
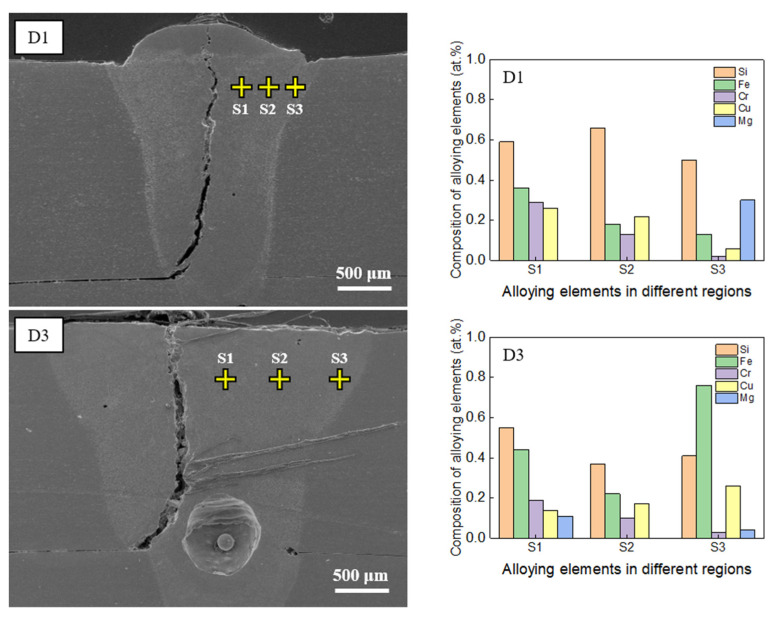
Results of energy-dispersive X-ray spectroscopy (EDS) point mapping in different locations without cracks for samples D1 and D3.

Figure 8 presents the EDS point-mapping results for samples D1 and D3 in the regions without hot cracking. The analysis showed that the concentrations of the major alloying elements Si, Fe, Cr, and Cu were lower in regions without cracks than those near cracks (as seen in Figure 7), indicating the pivotal role of the chemical composition in hot-crack formation. Although traces of Mg were detected in spectrum S3 for sample D1 and in spectra S1 and S3 for sample D3, the amount of Mg present was minimal. This uneven distribution of Mg in the weld zone can be attributed to several factors, including the vaporization of Mg due to its low boiling point and its segregation or migration near grain boundaries during solidification. As such, a non-uniform distribution of Mg within the weld zone is expected.

**Figure 9:**
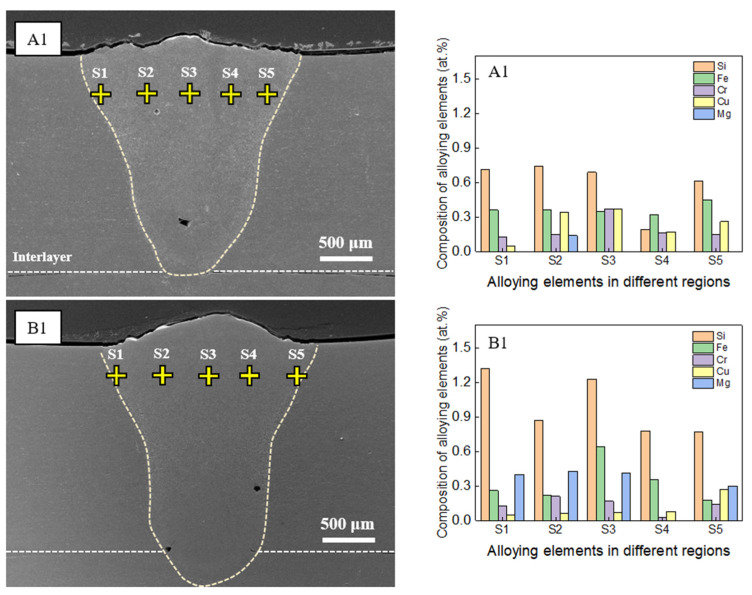
Results of elemental point mapping by energy-dispersive X-ray spectroscopy (EDS) for samples A1 and B1.

Figure 9 illustrates the results of the EDS point-mapping analysis for the samples that had a low heat input (30 J/mm) and were devoid of hot cracking. The analysis indicated that, for sample A1, the atomic percentages (at. %) of major alloying elements such as Si, Fe, and Cr, excluding Cu and Mg, were less than those of Si, Fe, and Cr in sample D1 (refer to Figure 7, crack region). The diminished concentrations of Cu and Mg in sample D1 were likely because of their vaporization caused by the high temperature in the weld zone, given that sample D1 had the highest laser power (2000 W). This explanation is consistent with the findings for sample B1, where the at. % of Si, Fe, and Cr was also lower than that in sample D1, excluding Cu and Mg. As shown in Figure 9, a considerable Mg concentration was found in the weld zone of sample B1 without hot cracking. According to Wang et al. [22], a high Mg concentration induced a low hot-cracking susceptibility during the directed energy deposition of an Al-Zn-Mg-Cu alloy. Thus, it is considered that the high concentration of Mg contributed to a sound weld formation in the case of sample B1.

As per the American Society for Testing and Materials standards, the permissible thresholds for alloying elements during the welding of AA6061, which minimize the propensity for hot cracking, are outlined in Table 3 [23].

Based on the analysis discussed in this section, the heightened vulnerability to hot cracking during the laser welding of AA6061 in a lap joint configuration is linked to high atomic concentrations of Si (ranging from 1.38% to 2.16%), Cr (ranging from 0.76% to 2.16%), and Fe (ranging from 0.69% to 1.11%). The concentrations of these elements in the region prone to cracking significantly exceeded the permissible limits outlined in Table 3, which prescribes general percentage ranges for these alloying elements to minimize the occurrence of hot cracking during welding. Notably, these limits fluctuate depending on the joint configuration and specific welding process parameters. The EDS analysis results suggest that surpassing the composition limits of Si, Cr, and Fe by 2.3, 8.1, and 2.7 times on average, respectively, can increase the risk of hot cracking during the laser welding of AA6061 in a lap joint configuration. Consequently, meticulous attention and control over the levels of these elements during the welding process are strongly recommended to mitigate the likelihood of hot cracking.

### 3.4. Mechanism of Hot Cracking

The mechanism of hot cracking in the laser welding of AA6061 is primarily determined by various factors such as the properties of the material, the welding parameters, and the joint design. In this study, we propose a mechanism to explain the hot-cracking phenomena. This mechanism, discussing the initiation and growth stages of hot cracking, is detailed in two separate subsections.

#### 3.4.1. Crack Initiation Mechanism

Figure 5 depicts the microstructure of the transverse cross-section of the WZ, composed of columnar and equiaxed grains—a typical structure frequently observed in Al alloys. This is attributed to the high-speed welding process, which results in rapid heat transfer to the base metal. The microstructure of the weld, as illustrated in Figure 5, features columnar grains near the fusion line. In this study, hot cracking was initiated from the root of the weld at the faying interface between the two plates, with the interface serving as a natural pre-crack [24]. This implies that a microcrack was already present at the welding onset.

**Figure 10:**
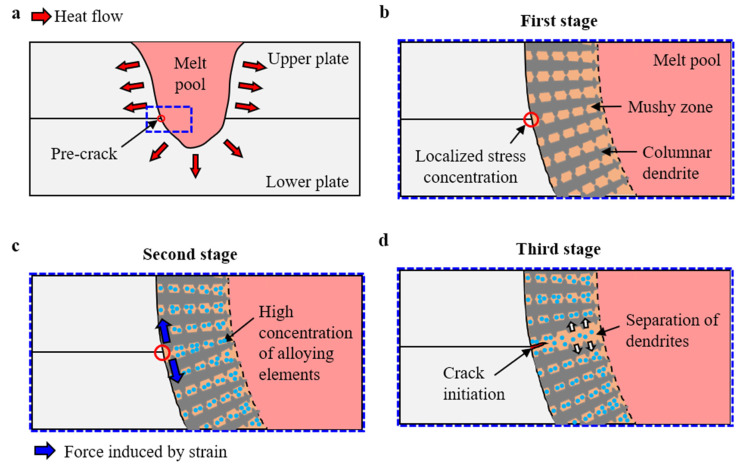
Schematic illustration of crack initiation in the weld zone: (**a**) lap joint configuration with a melt pool; (**b**) localized stress concentration at the interface; (**c**) strain induced between two dendrites due to highly coherent network; (**d**) crack initiation due to separation of dendrites.

Figure 10 illustrates a schematic of the crack initiation during the weld pool solidification at various stages. The lap joint configuration with a liquid melt pool is presented in Figure 10a. The pre-crack zone is indicated by a red circle in the diagram. The cooling process, which includes solidification, can be categorized into three stages (see Figure 10b–d).

In the initial stage, columnar grains primarily form, owing to the temperature gradient and the conditions of solidification experienced by the materials during the process. A higher temperature gradient and slower solidification rate near the fusion boundary encourage the growth of columnar grains. As the heat of the laser beam causes the materials to melt, a melt pool is created. When this pool solidifies, it generates tensile stress due to the shrinkage of the FZ. This stress tends to concentrate at the weld root of the faying interface between the two welding specimens. This concentration occurs because of a sudden geometry change at the weld root, disrupting the stress flow through the material and causing the weld root of the faying interface to act as a stress raiser. Consequently, the stress at the faying interface becomes notably higher than in the surrounding areas, leading to a concentration of stress.

During the solidification of the melt pool, the molten metal transitions from a liquid to a solid phase. As the temperature decreases, columnar dendritic structures start forming at the fusion boundary, extending into the remaining liquid (Figure 10b). Between these columnar dendrites, a mushy zone exists, where both the liquid and solid phases coexist. The width of the mushy zone depends on the freezing range of the alloy [25]. As Al alloys are wide-freezing-range alloys, they possess a relatively broad mushy zone [26]. This expansive mushy zone allows the dendrites of the Al alloys to grow uniformly, forming a coherent network, as shown in Figure 10b. The term coherency of the dendritic network refers to the uniform interconnection of the dendrites.

In the following stage, as illustrated in Figure 10c, the highly coherent network induces strain between two columnar dendrites due to their competing growth for the same space. As a result, the dendrites exert pressure on each other, creating a tendency to separate. As previously discussed, the faying interface at the fusion boundary functions as a pre-crack, leading to stress concentration at the weld root. Moreover, strain occurs between the two dendrites, rendering the weld root at the faying interface susceptible to hot cracking (Figure 10c). Concurrently, the alloying elements (Si, Fe, Cr, and Cu) are trapped in the interdendritic region. Given their low solubility and diffusivity in Al, these alloying elements do not readily blend with Al and segregate in the mushy zone. This segregation behavior results in the trapping of these elements between the dendrites during the rapid solidification of Al, leading to a high concentration of these elements in the remaining mushy zone.

Figure 10d illustrates the final stage of crack initiation. Owing to the strain generated between dendrites, these structures exert pressure on each other, as previously mentioned. The segregation of alloying elements within the mushy zone weakens the cohesive force between neighboring dendrites, facilitating their separation. Furthermore, the stress concentration at the weld root of the faying interface provides a conducive environment for the dendritic separation. As a result, crack initiation commences from the weld root of the faying interface, as depicted in Figure 10d.

#### 3.4.2. Crack Growth Mechanism

**Figure 11:**
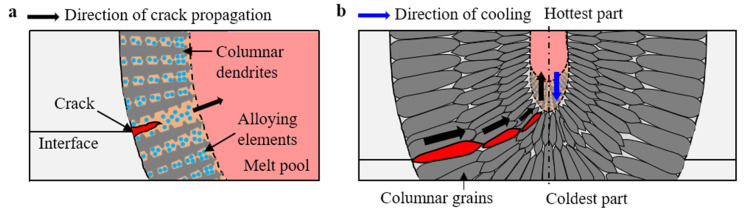
Schematic illustration of crack growth in the weld zone: (**a**) growth of hot crack due to interdendritic separation; (**b**) crack propagation in the FZ.

Figure 11 presents a schematic illustration of crack growth. Once intergranular hot cracking commences through the solidification of the dendritic structure, hot cracks start to propagate. At this stage, the alloying-element-rich liquid phase in the mushy zone weakens the cohesive force between neighboring dendrites, thus enabling their separation. This leads to inter-dendritic separation, as discussed in Section 3.4.1. When the dendrites separate from each other, the spacing between them increases, promoting the further growth of the intergranular hot cracks (see Figure 11a). This intergranular region of the columnar dendrites provides a path for hot-crack propagation. Consequently, crack propagation tends to align with the growth direction of the columnar dendrites, as illustrated in Figure 11b. When the crack reaches the central region of the FZ, it begins to propagate upward. Solidification generally occurs in the direction opposite to cooling. During cooling, heat dissipates from the top surface (the hottest part of the FZ) to the fusion boundary (the coldest part of the FZ). In the central area of the fusion zone, the direction of cooling is towards the bottom of the fusion zone. As a result, solidification and crack propagation in the central area of the FZ proceed upward. This crack propagation persists until the driving forces (such as a high stress, a high strain, or a high concentration of alloying elements in the intergranular region) for crack growth diminish.

## 4. Conclusions

In this study, we examined the laser welding of AA6061 plates in a lap joint configuration. We explored the influence of welding parameters, including the laser power and welding speed, on hot cracking, the weld microstructure, and the concentration of alloying elements. This investigation facilitated the establishment of a hot-cracking mechanism for laser-welded AA6061 in a lap joint configuration. The primary conclusions are as follows:

The study demonstrated that specimens exposed to a high heat input (50 J/mm) exhibited substantial hot cracking, while those exposed to a moderate heat input (40 J/mm) showed minor hot cracking. In contrast, specimens exposed to a low heat input (30 J/mm) did not exhibit hot cracking, except for the specimen exposed to the highest laser power (2000 W). Generally, a decrease in the heat input corresponded to a reduction in the hot-cracking susceptibility.

Specimens with coarse grain structures showed significant hot cracking compared to those with finer grain structures. This study also noted that specimens exposed to a high heat input exhibited a coarse-grained structure, whereas those exposed to a low heat input had a finer-grained structure.

The hot-cracked regions showed significantly higher concentrations of alloying elements than the acceptable limits for AA6061 (Si: 2.3 times, Cr: 8.1 times, and Fe: 2.7 times, on average). The chances of hot cracking in welds may increase if the concentrations of these elements surpass these limits. As such, controlling the elemental composition is recommended to minimize the risk of hot cracking.

Hot-cracking initiation occurred at the weld root of the faying interface between the two specimens. We discovered that the initiation of hot cracking was influenced by various factors. These factors include the geometrical features of the faying interface acting as a pre-crack surface, strain development between dendrites resulting from a coherent network, and a high concentration of alloying elements reducing the cohesive force between dendrites. As a result, cracks are initiated at the faying interface located at the fusion boundary.

Regarding the crack growth mechanism, the crack initiated from the weld root at the faying interface and propagated along the growth direction of the columnar dendrites. This is because the intergranular region serves as a crack propagation path. As the crack reached the central region of the FZ, it propagated upward due to the cooling direction in the center area of the FZ. This crack propagation continued until the driving force for the crack diminished.

As mentioned above, samples with a high heat input or laser power condition were more vulnerable to hot cracking. Under these conditions, a higher melt pool temperature and more rapid changes in the temperature are induced. Accordingly, the likelihood of hot-cracking generation increases. In order to improve the hot-cracking issue in the laser welding of Al alloys, using dual-core fiber laser or dual-beam laser welding (using additional pre- or post-heating laser beams), which can provide gentle variation in the melt pool temperature, is considered a good approach.

## Figures and Tables

**Table 1:** Chemical composition of AA6061.

Material	Al	Si	Fe	Cu	Mn	Mg	Cr	Zn	Ti	Others
wt. %	97.34	0.71	0.5	0.24	0.12	0.9	0.18	0.08	0.04	0.01
at. %	97.78	0.68	0.24	0.11	0.05	1.0	0.08	0.03	0.03	0.01

**Table 2:** Laser welding process conditions.

Exp. No.	Laser Power (W)	Welding Speed (mm/s)	Heat Input (J/mm)	Power Density (×10^5^ W/mm^2^)	Interaction Time * (×10^−3^ s)
A1	1400	47	30	1.78	2.13
A2		35	40		2.86
A3		28	50		3.57
B1	1600	53	30	2.03	1.89
B2		40	40		2.50
B3		32	50		3.13
C1	1800	60	30	2.29	1.67
C2		45	40		2.22
C3		36	50		2.78
D1	2000	67	30	2.54	1.49
D2		50	40		2.00
D3		40	50		2.50

* Interaction time: beam spot size/welding speed.

**Table 3:** Acceptable limit for alloying elements in AA6061.

Alloying Element	wt. %	at. %
Si	0.4–0.8	0.38–0.77
Fe	0.7	0.33
Cu	0.15–0.4	0.06–0.17
Mn	0.15	0.07
Mg	0.8–1.2	0.89–1.33
Cr	0.04–0.35	0.02–0.18
Zn	0.25	0.10
Ti	0.15	0.08
Al	97.16	97.59
others	0.2	0.48

## Data Availability

The data will be made available upon request.
